# Early contraceptive implants removal and its associated factors among women using implants at a National Referral Hospital, Kampala Uganda

**DOI:** 10.1186/s12905-021-01541-9

**Published:** 2021-12-01

**Authors:** Gerald Ssebatta, Dan Kabonge Kaye, Scovia Nalugo Mbalinda

**Affiliations:** 1 Department of Nursing, College of Health Sciences, Makerere University, P.O. Box 7072, Kampala, Uganda; 2 Department of Obstetrics and Gynecology, College of Health Sciences, Makerere University, P.O. Box 7072, Kampala, Uganda

**Keywords:** Early implant removal, Implant, Contraception

## Abstract

**Background:**

Early discontinuation of implant contraceptive methods and reasons for discontinuation remains a major concern for family planning programs. Early discontinuation is related to higher rates of the overall fertility rate, unwanted pregnancies leading to possibly induced abortion. There is paucity of data on the practice of discontinuation of contraceptives in developing countries. The objective of the study was to determine the magnitude of early implants discontinuation among women receiving implants services in the study area and the factors associated with it.

**Methods:**

A cross-sectional study was conducted from 2nd January to 3rd March 2020. Data were collected from 207 women who had come to remove implants on socio-demographic characteristics, obstetric history, duration of implant, and reasons for wanting to remove the implant. We computed the proportion of those who removed the implant before 18 months (early discontinuation). To assess the factors associated with early discontinuation, we estimated the prevalence ratios with a generalized linear model of the poisson family with a log link and robust error variance.

**Results:**

The proportion of early implant discontinuation was 87/207(42%). Factor associated with early implant discontinuation included; experience of side effects (PR = 1.1; 95% CI 1.03–1.24; *P* = 0.001), not having received pre-insertion counseling about the benefits and side effects of contraceptive implants (PR = 1.5; 95% CI 1.02–1.30; *P* = 0.019) and staying in rural areas (PR = 1.1; 95% CI 1.03–1.27; *P* = 0.014).

**Conclusion:**

Nearly one in every two mothers have early discontinuation of contraceptive implants. Factors associated with early implant removal include; experience of side effects, lack of counseling services, and staying in rural areas. There is a need for intervention to address high prevalence of early contraceptive removal through improving on counselling services about possible side effects.

## Background

According to the Uganda Demographic Health Survey, 2016, Uganda has a high fertility rate of 5.4 which is the highest in East Africa. Uganda family planning costed plan, 2014 noted a high number of unplanned pregnancies amongst rural, poor, and less educated. Also, a higher 32% of sexually active unmarried compared to 28% of currently married women have an unmet need for family planning. The use of implants alone is as low as 6% among married women [1]. The unmet need for contraception among married women worldwide is 10.7% [2]. More than 200 million women around the world have an unmet need for contraception [3]. Although contraception improves women's life and public health, many clients discontinue contraceptive use because of dissatisfaction related to side effects, contraceptive failure or other factors [4]. This hinders family planning 2020 aim of having an additional 120 million women and adolescents receiving family planning services by 2020 [5].

Contraceptive discontinuation is a worldwide problem that is associated with a high number of unplanned pregnancies and in a year, 182 million pregnancies across the world are unplanned that can be related to improper usage of contraceptives [6]. Implants that have been available since 1998 [7], are long-acting progestogen-only reversible contraceptive subdermal rods that act by inhibiting ovulation [8]. The method is highly effective with a failure rate of less than 1% in a year [9]. It is easy to insert and remove though requires a qualified health care provider [10]. Once the women are inserted, they require little user compliance and there is rapid return of fertility immediately after removal [8].

There are three types of contraceptive implants that include Implanon and Nexplanon, Sino-implant and Jadelle [11]. Implanon NXT is a single-rod long-acting subdermal hormonal contraceptive for 3-years of use, containing 68 mg etonogestrel [12]. It is made of ethylene–vinyl acetate copolymer with a length of 40 mm and a diameter of 2 mm [13]. Jadelle is a set of two flexible cylindrical implants consisting of dimethylsiloxane/methyl vinyl siloxane copolymer core enclosed in thin-walled silicone tubing [14]. Each rod contains 75 mg of levonorgestrel and is licensed for use for 5 years [15, 16]. After the implant has been inserted there is no need for frequent visits to family planning clinics though it is associated with changes in the bleeding pattern [7].

The main consequence of early removal of implants is unwanted pregnancy that results in unsafe abortion, inadequate antenatal care, adolescent pregnancy, and socioeconomic and health disparities [17]. These unintended pregnancies are associated with high rates of mortality and morbidity among women of reproductive age [18].

The reasons for removal before the recommended time were not recorded and are unknown. Currently, Kawempe NRH provides Implanon NXT and Jadelle to women for contraception as Long Acting Reversible Contraceptive (LARC) method of family planning which are advocated for by the Ministry of health (MoH). There is a need to understand why women have early removal of contraceptive implants in response to achieving the Sustainable Development Goals especially Goal 3 which ensures healthy lives and promoting wellbeing for all of all ages [19]. There is limited research in Uganda and Kawempe in particular on why women have early implants removal and factors associated with it. The study findings will therefore enlighten the gaps which can be solved by health workers and stakeholders to improve clients' response to family planning services.

Efforts to increase academic attainment, effective counseling on the side effects at the time of insertion, and follow up of women will impact the continuation of implants positively [6]. Also, public enlightenment on contraceptive efficacy and safety will improve its acceptance among illiterate women [14].

## Methodology

### Study setting

The study was conducted at the family planning clinic, Kawempe NRH in Kampala Uganda. Kawempe NRH is, 4.2 km away from Kampala City Center. It is a government facility that provides maternal and child health services to the public including family planning services has a bed capacity of 1,500 beds According to secondary data collected from the family planning clinic at Kawempe NRH showed that 235 out of 625 women who were using implants between 22/10/2018 and 6/02/19 opted to discontinue but the duration of implant usage and reasons for removal were not recorded.

### Study design

A facility-based cross-sectional study was conducted among women above 18–49 years who had used contraceptive implants and requested for removal at Kawempe National Referral Hospital. Data were collected for almost 2 months from 2nd-January to 4th-March 2020. Every woman was interviewed on the same day she discontinued the implant and the date on which she inserted the implant was recorded from the client card that had been given to her after insertion, this minimized recall bias.

### Data collection

Data were collected once from each participant using the quantitative. Data were collected on socio-demographic (age, marital status, religion, occupation, and education), obstetric (number of children, counseling, past contraception utilization), for those who had used the implant were asked whether they had experienced side effects like menstrual disruption, acne, and arm pain etc., whether they came back to the family planning clinic after experiencing the side effects, and also asked whether they had used any modern contraceptive before. Women were also asked if their spouses and peers had an influence in implant usage. Data was collected on reasons to why they were removing implant that included pattern opposition, experience of side effects, peer influence and so on.

### Sample size determination

The sample size was determined by using the desired precision e = 0.05 for the prevalence of early discontinuation at Kawempe NRH, a confidence level of 95% and z- statistic for the level of confidence Z = 1.96. Using an equation developed by (Glenn, 1992) [20] for calculating the sample size in research studies, a sample size of 207 respondents was used in the study with a 100% response.

Using equation developed by (Glenn 1992) [20], **n**_**o**_** = Z**^**2**^**pq/e**^**2**^, where n_o_ is the sample size, Z^2^ is the abscissa of the normal curve that cuts off an area alpha at tails (1-alpha equals the desired confidence level, e.g., 95%), P is the estimated proportion of an attribute that is present in the population, e is the desired level of precision and q is (1 − p). The value for Z is found in statistical tables that contain the area under the normal curve. Therefore, if the estimated prevalence of early implants removal, p = 16% [21]. The sample size will equal to 207 women.

### Sampling procedure

The family planning clinic opened at 8:00 am and closed at 5:00 pm from Monday to Friday, and all women were health educated and counselled about contraception use at 11:00 am. After counselling session, women who had come to discontinue the implant were gathered in one place in the linear order as they sat on the bench. We explained the purpose, benefits and risks of participation regarding this study and those who consented were included. Women were asked whether they had carried their implant card, and only those who had it were eligible for the study. Those who accepted were called to a private room to sign the consent form. The first participant who arrived at the health facility was selected to be the first and every the other person in the line were included in the study. Questionnaires were administered to them and after answering it fully, they proceeded to the procedure room.

### Inclusion criteria

I. The study was composed of women 18 years and above who had come to discontinued implants at family planning clinic, Kawempe NRH during the study period.
II. For a respondent to be included in the study they must have been a visible record of the date of insertion of the contraceptive implant, either as a client card.

### Data collection instruments and definition of variables

Data were collected using a semi-structured face-to-face questionnaire having three parts. The first part contained the socio-demographic characteristics (age, marital status, religion, occupation, education, pattern opposition and involvement, peer influence). The second part of the questionnaire consisted of obstetric characteristics (number of children, and abortion) while the third part consisted of method-related characteristics(side effects, follow-up, conception, counseling, past contraception utilization).

Dependent variable; Early implants removal was the dependent variable, defined as discontinuation at 18 months or less after implant insertion [22]. It was measured as a binary variable coded as ‘Yes’ if an implant was discontinued before 18 months from the time of insertion and ‘No’ if 18 or more months.

Independent variables; these included; socio-demographic factors (age, marital status, religion, occupation, and education), obstetric factors (number of children and number of induced abortion), social factors (husband perception and peer group influence) and method related factors (type and duration of usage, side effects, follow up, conception, counseling, past contraception utilization).

### Data collection procedure

Participants were recruited at the family planning clinic, Kawempe NRH, every Monday to Friday, between 8.00 am and 4.00 pm.

There was one research assistant who was trained for one day before data collection on the contents of the study, contents of the questionnaires, and interview skills.

### Data analysis and management

The raw data from the filled questionnaires were entered into Statistical Package for Social Services (SPSS), cleaned, coded. Descriptive statistics including proportion, percentage, mean, median, and standard deviation were presented in text and tables. Results of Bivariate and multivariate analysis are presented in table. To assess the factors associated with early implant removal, we estimated the prevalence ratios (RR) with a generalized linear model of the poisson family with a log link and robust error variance.

The significance level was set at 5% for all tests of significance.

## Results

Table 1 shows the socio-demographic characteristics of the respondents. Most participants 140/207 (67.6%) were aged 25 years and above, 128/207 (61.8%) had attained secondary level or above, 158/207 (76.3%) were married, 82/207 (39.6%) were Catholics, while 153/207 (73.9%) stayed in urban area. The participants were also asked about occupation majority, 87 (42.0%) were housewives, 60 (29.0%) were employed, 56(27.1%) were traders and majority of their spouses 92 (44.5%) were traders (Table 1).

**Table 1:** Social-demographic, obstetric history, contraceptive related characteristics of implants users at National Referral Hospital, Kampala Uganda (n = 207)

Variable	Frequency	Percentage (%)
Social-demographic		
Woman's age		
18–24	67	32.4
25 and above	140	67.6
Marital status		
Divorced	5	2.4
Cohabiting	16	7.7
Widowed	3	1.4
Married	158	76.3
Single	25	12.1
Religion		
Catholic	82	39.6
Born again	54	26.1
Protestant	46	22.2
Muslim	19	9.2
Jehova	6	2.9
Address		
Urban	153	73.9
Rural	54	26.1
Level of education		
Below secondary	79	38.2
Secondary and above	128	61.8
Women’s occupation		
Housewife	87	42.0
Employee	60	29.0
Trader	56	27.1
Student	4	1.9
Obstetric history		
Number of living children		
0–2	130	62.8
3+	77	37.2
History of abortion		
Yes	69	33.3
No	138	66.7
The desire for more children in the future		
Yes	165	79.7
No	42	20.3
Ever used modern contraceptive before insertion of Implants		
Yes	135	65.2
No	72	34.8
Type of contraceptive used before insertion of Implants (n = 135)		
Injectable	85	63.0
Condoms, IUCD, OCP	50	37.0
Type of implant used		
Implanon NXT	145	70.0
Jadelle	62	30.0
Experience of side effects		
Yes	174	84.1
No	33	15.9
Counseling about efficacy and side effects		
Yes	170	82.1
No	37	17.9
Had follow-up appointments		
Yes	90	43.5
No	117	56.5

### Obstetrics related characteristics

The majority of participants 121/207 (58.5%) had at least 1–2 children while 13/207 (6.3%) had more than five alive children. Participants were also asked if they had ever had an abortion, 69/207 (33.3%) had ever had an abortion but the majority had ever had it once 52/69 (75.4%). One hundred sixty-five (79.7%) had a desire for more children in the future, out of those who had a future desire to have children 95/165 (57.6%) wanted to have babies after 2 years while 56/165 (33.9%) wanted to have babies in less than one year (Table 1).

Table 1 also shows results of method related characteristics and majority of the participants 135/207 (65.2%) had ever used modern contraceptives before the currently discontinued implant, out of those who had used modern contraceptives before 85/135 (63.0%) were using injectable followed by oral contraceptive pills 20/135 (14.8%). Most participants 145/207 (70.0%) were using Implanon NXT while 62/207 (30.0%) were using Jadelle. All the participants were asked whether they received counselling about the implants in terms of efficacy and side effects, whereby 170/207 (82.1%) reported that they were counseled. Then further asked whether after insertion of implants they were given future appointment dates, 117/207 (56.5%) of the study participants did not have appointments follow-up during their implant utilization period (Table 1).

The proportion of early discontinuation of implants was 87/207 (42.0%). All of the women who removed the implants due to the desire to conceive 63/207 (30.4%), and those who switched to another method 12/207 (5.8%) discontinued before 18 months. For compensation of early implant removal, one would prefer women to discontinue and switch or to discontinue in order to conceive. Therefore, a target proportion can be determined by ((sum of (women who discontinue early and switch = 12 plus women who discontinue early to conceive = 63)/by total of women who discontinue early = 87)) of 0.42 equivalent to 36.2% (Table 2).

**Table 2:** Proportion of women of reproductive age 15–49 years who discontinued implants early at a National Referral Hospital (n = 207)

Variable	Frequency	Percentage (%)
Early discontinuation of implants (n = 207)		
Yes	87	42.0
No	120	58.0
Early discontinuation of implanon NXT (n = 145)		
Yes	64	44.1
No	81	55.9
Early discontinuation of Jadelle (n = 62)		
Yes	23	37.1
No	39	62.9
Min. duration of use (1 month)	9	4.3
Min. duration of use (60 months)	8	3.9
Median duration of use	19	
Early removal due to desire to conceive (n = 63)		
Yes	63	100
No	0	0
Early removal due to switching to another method (n = 12)		
Yes	12	100
No	0	0

The major reason for early removal of implants was due to side effects that accounted for 174/207 (84.1%) that included 139/174 (54.5%) for menstrual disruption, 56/174 (20.4%) for headache, weight body changes 43/174 (15.6%), insertion site pain 21/174 (7.64%), acne and pruritus 9/174 (3.3%), and loss of sexual desire 7/174 (2.6%) (Table 3). These were followed by 63/207 (30.4%) those participants who had the plan to conceive shortly, 42/207 (20.3%) had been influenced by their partners to discontinue, 6/207 (2.9%) claimed that they had conceived while using implant but one participant was interviewed and was found to be pregnant before implant insertion (Table 4).

**Table 3:** The main side effects of Implant for early discontinuation among women using implants at Kawempe NRH (n = 174)

Side effects	Frequency	Percentage (%)
Menstrual disruption	139	50.54
Headache	56	20.36
Weight gain/loss	43	15.64
Arm pain/insertion site pain	21	7.64
Acne and pruritus	9	3.27
Loss of sexual desires	7	2.55
Total	275	100

NB: Most of the women reported more than one side effects

**Table 4:** Reasons for removal of the contraceptive implant at National Referral Hospital, Kampala Uganda (n = 207)

Reasons for removal	Frequency	Percentage (%)
Desire for pregnancy	63	16.9
Partner influence	42	11.3
Husband’s death	4	1.1
Switch to another method	12	3.2
Divorce	5	1.3
Side effects	174	46.6
Traveling abroad	18	4.8
Conception	6	1.6
Expiry	49	13.1
Total	373	100

NB: Women gave more than one reason for discontinuation of implant

### Factors associated with early implants removal among women using implants at NRH

The early discontinuation rate of implants was 87/207 (42%) using a cut-off of 18 months. Table 5 shows factors associated with early contraceptive implant discontinuation. No variable was excluded from analysis for the association because each category had desired frequencies when checked by cross-tabulation.

**Table 5:** Factors associated with early implants removal among women using implants at NRH (n = 207)

Variables	PR (95% CI)	*P*-value	A. PR (95% CI)	*P*-value
Age of participants				
18–29	1.076 (0.987–1.173)	0.097	1.079 (0.995–1.170)	0.066
> 29	1			
Counseling about benefits and side effects				
Yes	1		1	
No	1.51 (1.02–1.3)	0.023*	1.54 (1.02–1.30)	0.019*
Abortion history				
Yes	1		1	
No	1.1 (0.98–1.17)	0.125	1.04 (0.95–1.13)	0.422
Removal due to side effects				
Yes	1.137 (1.033–1.252)	0.009*	1.131 (1.023–1.242)	
No	1		1	0.010*
Address of participants				
Urban	1		1	
Rural	1.15 (1.033–1.271)	0.010*	1.14 (1.03–1.27)	0.014*

NB: *P < 0.05: significant variables; *PR* prevalence ratio, *A.PR* adjusted prevalence ratio

The results showed that women who had experienced side effects discontinued the implant early (PR = 1.13; 95% CI 1.02–1.24, *P* = 0.010). Women who had not been counseled removed implant early (PR = 1.5; 95% CI 1.02–1.304, *P* = 0.019). Women who resided in rural areas removed implant early (PR = 1.1; 95% CI 1.03–1.27, *P* = 0.014) (Table 5).

## Discussion

The study established the factors associated with early implant removal among women at a National Referral Hospital aged 18–49 years. The proportion of early implant removal was high, and factors associated with it were the experience of side effects, lack of effective counseling before insertion, and staying in rural areas.

### Proportion of early discontinuation of implants

This facility-based study assessed for early discontinuation of implants (Implanon and Jadelle) among women who were using implants at Kawempe NRH, Kampala district. The prevalence of early discontinuation of implants in this study is similar to that of a study done in Sudan which found a discontinuation rate of 43% [23]. However, the current proportion is higher than other studies conducted as in Ethiopia 16% [21] and 23.4% [8]. Other studies done in Ethiopia had a discontinuation rate higher than the current study. In a study conducted in Debre Tabor Town, Northwest Ethiopia, early discontinuation was at 65% [6]. The difference could be attributed to the improved effort of pre-insertion counseling particularly about the possible side effects of the method as compared to the previous study. In the previous study in Debre Tabor Town, 31.8% were counseled about the possible side effects compared to the current study were 82.1% were counseled [6]. Also, more than half in the current study had ever used any other modern contraceptive method before, this increases the chance of a continuation of implants since participants might have familiarized themselves with side effects of contraceptive methods [24].

Different studies show variations in discontinuation rates. This could be attributed to different cut off for early discontinuation whereby in Ethiopia 2.5 years was used as cut off for early implant removal [6], while a cut of 18 months was used in Uganda [22]. In other studies, the low discontinuation rates were attributed to effective pre-insertion counselling about possible side effects. For instance, in a study conducted in Mekelle City 34 percent of the women who were counselled discontinued early while in the current study 38.2 percent discontinued early of the women who were counselled [23]. In Uganda, there is a study that assessed early discontinuation of both contraceptive implants with an early discontinuation rate was 31% in the facility-based cross-sectional study conducted in the Wakiso District. This was lower compared to the current study and the difference could be attributed to the denominator for assessing the proportion whereby the current study only included age of 18 years and above while the study done in Wakiso district included women of reproductive age 15–49 years from 42 health facilities in a rural and urban setting [22]. The other reason for the difference could be attributed to a cost incurred to retain the implant where 27.2% of women incurred a cost in the previous study conducted in Wakiso district compared to the current study where services rendered to women were for free of charge, this would be a barrier from seeking health care whenever they experience side effects.

Pre-insertion counselling in family planning helps women and their partners to gain increased control over their reproductive health. Providing counseling during the insertion of Implants was positively associated with the continuation of use [8]. Preplacement counseling about the possible side effects of the method and support by the service providers might be the most important way to help women continue on Implants contraception [25]. Women and couples who receive better counseling on their method may be more aware of potential side effects and how to cope with them. In this study, the women who were not counselled were more likely to have early implant removal. This is consistent with the study findings conducted in Debre Tabor Town, Northwest Ethiopia [6]. This is because when the women are informed about the possible side effect of the method, they will tolerate side effects but those who were not informed will seek the removal of the method. Timely follow up and appropriate counseling helps the women to cope with side effects which ultimately prevents discontinuation. The acceptance and awareness of any contraceptive implants will be increased through involving the couple during counseling sessions [26].

The occurrence of side effects has a negative impact on whether a woman continues to use family planning. In this study women who experienced side effects were more likely to have early implant removal. This is consistent with the study conducted in Tigray [21] and Debre Tabor Town [6] were women who experienced a side effect of the method led to early Implant discontinuation. In addition to this, respondents may fear different complication as a result of side effects, this may lead to discontinuation of implant early. The main reasons for the removal of the implant in this current study were side effects, followed by a desire for more children, expiry, partner influence, and conception. Among the side effects, menstrual disruption and headaches were most common. This is consistent with other studies conducted in Ethiopia [21]. Menstrual disruption has no serious effects on health but can interfere with daily activities, especially sexual relationships and possibly with work [21]. Women who requested implant removal due to side effects would have received misleading information from non-professional persons in the community, lacked appropriate counseling about side effects and appointment dates for follow-up. This means that immediate action upon the management of bleeding problems will enable the continuation of the contraceptive implant.

One of the reasons for early implant removal was pressure from spouses. Men need to understand their role in reproduction so that they can share the responsibility for family planning and birth spacing. In this study partner disapproval of the contraceptive implant was among the reasons for the discontinuation and accounted for a fifth of early implant removal. Male involvement in maternal health contributes a lot to the reduction of mortality and improvement of maternal health as well as family health [9]. This suggests that to enable the continuation of implants, males should be involved in counseling sessions concerning contraception, before and during the period of implant use.

Residence appeared to be one factor associated with early removal of implants. Women in urban areas may be of better socioeconomic status, easy access to family planning services, cultural disparity, and high level of female literacy as compared to women in rural areas. This study showed that women who live in urban areas were less likely to discontinue implants early compared to those who live in rural areas. The difference can be attributed to occupation status. It is possible that women who live in rural areas are housewives and may discontinue implants early to have more children compared to their counterparts in urban areas who work in different jobs. This is in line with a study conducted in Dale District, Southern Ethiopia where women with high monthly income were less likely to discontinue the method [8]. Women in urban areas might have attained a higher level of education who often seek early healthcare whenever there is any disturbance from normal physiology [27] this increased number of encounter with health workers promotes confidence among women. Also, women who have attained a higher level of education can get more information from different reliable sources about contraceptive implants [23].

### Limitation

Being a cross-sectional study that was conducted from a health facility, it was not easy to determine the impact of early discontinuation of implants. Though the data collector was a midwife and well trained before data collection, she was a staff at that health facility, so the interview's bias would not be excluded from this study.

## Conclusions

In this study discontinuation of implants was high as nearly one of every 2 women had early removal of the implant. Experience of side effects, counseling services, and staying in rural areas were factors associated with early discontinuation. Quality family planning services that involve males during counseling sessions on the benefits and side effects are a vital way through which early implants discontinuation can be minimized.

## Data Availability

The datasets used and/or analyzed during the current study are available from the corresponding author on reasonable request.

## Abbreviations

CI: Confidence interval
IUCD: Intrauterine contraceptive device
LARC: Long acting reversible contraceptive
MoH: Ministry of health
NRH: National Referral Hospital
OR: Odds ratio
OCP: Oral contraceptive pills
UBOS: Uganda Bureau of Statistics
UDHS: Uganda Demographic Health Survey
